# Supplementary material for: Mapping the evolution of fertility support policies in China: A content and instrumental analysis
Source: PLoS One. 2025 Oct 9;20(10):e0332137. doi: 10.1371/journal.pone.0332137 (PMC12510515; doi:10.1371/journal.pone.0332137)
Supplement: S1 Appendix — (ZIP) [file pone.0332137.s001.zip › S1 Appendix. 226 original policy documents/62-国务院办公厅关于印发生育保险和职工基本医疗保险合并实施试点方案的通知.doc]

国务院办公厅关于印发生育保险和职工基本医疗保险合并实施试点方案的通知

**发布部门：** [国务院办公厅](https://www.pkulaw.com/law/chl?Aggs.IssueDepartment=601) [机构沿革](javascript:void(0)) [C:\email\index](../../../../C:/email/index)

**发文字号：**国办发〔2017〕6号

**发布日期：**2017.01.19

**实施日期：**2017.01.19

**时效性：** [现行有效](https://www.pkulaw.com/law/chl?Aggs.TimelinessDic=01)

**效力级别：** [国务院规范性文件](https://www.pkulaw.com/law/chl?Aggs.EffectivenessDic=XC0203)

**法规类别：** [保险综合规定](https://www.pkulaw.com/law/chl?Aggs.Category=04402) [营商环境优化](https://dobusiness.pkulaw.com/)

国务院办公厅关于印发生育保险和职工基本医疗保险合并实施试点方案的通知
（国办发〔2017〕6号）

各省、自治区、直辖市人民政府，国务院各部委、各直属机构：
　　《生育保险和职工基本医疗保险合并实施试点方案》已经国务院同意，现印发给你们，请试点地区和各有关部门加强组织领导，认真贯彻执行。

国务院办公厅
2017年1月19日

生育保险和职工基本医疗保险
合并实施试点方案

　　为贯彻落实党的十八届五中全会精神和《[中华人民共和国国民经济和社会发展第十三个五年规划纲要](https://www.pkulaw.com/chl/3fd7b48d6cb8d951bdfb.html)》，根据《[全国人民代表大会常务委员会关于授权国务院在河北省邯郸市等12个试点城市行政区域暂时调整适用〈中华人民共和国社会保险法〉有关规定的决定](https://www.pkulaw.com/chl/4da3e0c3c6d838b6bdfb.html)》，现就做好生育保险和职工基本医疗保险（以下统称两项保险）合并实施试点工作制定以下方案。
　　一、总体要求
　　（一）指导思想。全面贯彻党的十八大和十八届三中、四中、五中、六中全会精神，深入贯彻习近平总书记系列重要讲话精神和治国理政新理念新思想新战略，认真落实党中央、国务院决策部署，统筹推进“五位一体”总体布局和协调推进“四个全面”战略布局，牢固树立和贯彻落实创新、协调、绿色、开放、共享的发展理念，遵循保留险种、保障待遇、统一管理、降低成本的总体思路，推进两项保险合并实施，通过整合两项保险基金及管理资源，强化基金共济能力，提升管理综合效能，降低管理运行成本。
　　（二）主要目标。2017年6月底前启动试点，试点期限为一年左右。通过先行试点探索适应我国经济发展水平、优化保险管理资源、促进两项保险合并实施的制度体系和运行机制。

　　二、试点地区
　　根据实际情况和有关工作基础，在河北省邯郸市、山西省晋中市、辽宁省沈阳市、江苏省泰州市、安徽省合肥市、山东省威海市、河南省郑州市、湖南省岳阳市、广东省珠海市、重庆市、四川省内江市、云南省昆明市开展两项保险合并实施试点。未纳入试点地区不得自行开展试点工作。

　　三、试点内容
　　（一）统一参保登记。参加职工基本医疗保险的在职职工同步参加生育保险。实施过程中要完善参保范围，结合全民参保登记计划摸清底数，促进实现应保尽保。
　　（二）统一基金征缴和管理。生育保险基金并入职工基本医疗保险基金，统一征缴。试点期间，可按照用人单位参加生育保险和职工基本医疗保险的缴费比例之和确定新的用人单位职工基本医疗保险费率，个人不缴纳生育保险费。同时，根据职工基本医疗保险基金支出情况和生育待遇的需求，按照收支平衡的原则，建立职工基本医疗保险费率确定和调整机制。
　　职工基本医疗保险基金严格执行[社会保险基金财务制度](https://www.pkulaw.com/chl/e4e06e49beb752e2bdfb.html)，两项保险合并实施的统筹地区，不再单列生育保险基金收入，在职工基本医疗保险统筹基金待遇支出中设置生育待遇支出项目。探索建立健全基金风险预警机制，坚持基金收支运行情况公开，加强内部控制，强化基金行政监督和社会监督，确保基金安全运行。
　　（三）统一医疗服务管理。两项保险合并实施后实行统一定点医疗服务管理。医疗保险经办机构与定点医疗机构签订相关医疗服务协议时，要将生育医疗服务有关要求和指标增加到协议内容中，并充分利用协议管理，强化对生育医疗服务的监控。执行职工基本医疗保险、工伤保险、生育保险药品目录以及基本医疗保险诊疗项目和医疗服务设施范围。生育医疗费用原则上实行医疗保险经办机构与定点医疗机构直接结算。
　　（四）统一经办和信息服务。两项保险合并实施后，要统一经办管理，规范经办流程。生育保险经办管理统一由职工基本医疗保险经办机构负责，工作经费列入同级财政预算。充分利用医疗保险信息系统平台，实行信息系统一体化运行。原有生育保险医疗费结算平台可暂时保留，待条件成熟后并入医疗保险结算平台。完善统计信息系统，确保及时准确反映生育待遇享受人员、基金运行、待遇支付等方面情况。
　　（五）职工生育期间的生育保险待遇不变。生育保险待遇包括《[中华人民共和国社会保险法](https://www.pkulaw.com/chl/83649bdaef86fe17bdfb.html)》规定的生育医疗费用和生育津贴，所需资金从职工基本医疗保险基金中支付。生育津贴支付期限按照《[女职工劳动保护特别规定](https://www.pkulaw.com/chl/cdf82292dbb62ee9bdfb.html)》等法律法规规定的产假期限执行。

　　四、保障措施
　　（一）加强组织领导。两项保险合并实施是党中央、国务院作出的一项重要部署，也是推动建立更加公平更可持续社会保障制度的重要内容。试点城市所在省份要高度重视，加强领导，密切配合，推动试点工作有序进行。人力资源社会保障部、财政部、国家卫生计生委要会同有关方面加强对试点地区的工作指导，及时研究解决试点中的困难和问题。试点省份和有关部门要加强沟通协调，共同推进相关工作。
　　（二）精心组织实施。试点城市要高度重视两项保险合并实施工作，按照本试点方案确定的主要目标、试点措施等要求，根据当地生育保险和职工基本医疗保险参保人群差异、基金支付能力、待遇保障水平等因素进行综合分析和研究，周密设计试点实施方案，确保参保人员相关待遇不降低、基金收支平衡，保证平稳过渡。2017年6月底前各试点城市要制定试点实施方案并组织实施。
　　（三）加强政策宣传。试点城市要坚持正确的舆论导向，准确解读相关政策，大力宣传两项保险合并实施的重要意义，让社会公众充分了解合并实施不会影响参保人员享受相关待遇，且有利于提高基金共济能力、减轻用人单位事务性负担、提高管理效率，为推动两项保险合并实施创造良好的社会氛围。
　　（四）做好总结评估。各试点城市要及时总结经验，试点过程中发现的重要问题和有效做法请及时报送人力资源社会保障部、财政部、国家卫生计生委，为全面推开两项保险合并实施工作奠定基础。人力资源社会保障部、财政部、国家卫生计生委要对试点期间各项改革措施执行情况、实施效果、群众满意程度等内容进行全面总结评估，并向国务院报告。
